# Kv3 K^+^ currents contribute to spike-timing in dorsal cochlear nucleus principal cells

**DOI:** 10.1016/j.neuropharm.2018.02.004

**Published:** 2018-05-01

**Authors:** Timothy Olsen, Alberto Capurro, Nadia Pilati, Charles H. Large, Martine Hamann

**Affiliations:** a Department of Neuroscience, Psychology and Behaviour, University of Leicester, University Road, Leicester LE1 7RH, UK; b Autifony Srl, Via Ugo Bassi 58b, Universita' di Padova, 35131 Padova, Italy; c Autifony Therapeutics Ltd, Stevenage Bioscience Catalyst, Gunnels Wood Road, Stevenage, SG1 2FX, UK

**Keywords:** Action potential, Auditory brainstem, Dorsal cochlear nucleus, Kv3 K+ current, Spike-timing, Acoustic over-exposure, ACSF, artificial cerebrospinal fluid, AOE, acoustic over-exposure, AUT1, (5R)-5-ethyl-3-(6-((4-methyl-3-(methyloxy)phenyl)oxy)-3-pyridinyl)-2,4-imidazolidinedione, CI, correlation index, CR, coincidence ratio, CV, coefficient of variation, DCN, dorsal cochlear nucleus, FC, fusiform cell, GAB, gabazine, ISI, inter-spike interval, KYN, kynurenic acid, NBQX, 2,3-dihydroxy-6-nitro-7-sulfamoyl-benzo[f]quinoxaline-2,3-dione, PPI, pre-pulse inhibition, STR, strychnine, TEA, tetraethylammonium

## Abstract

Exposure to loud sound increases burst-firing of dorsal cochlear nucleus (DCN) fusiform cells in the auditory brainstem, which has been suggested to be an electrophysiological correlate of tinnitus. The altered activity of DCN fusiform cells may be due to down-regulation of high voltage-activated (Kv3-like) K^+^ currents. Whole cell current-clamp recordings were obtained from DCN fusiform cells in brain slices from P15-P18 CBA mice. We first studied whether acoustic over-exposure (performed at P15) or pharmacological inhibition of K^+^ currents with tetraethylamonium (TEA) affect fusiform cell action potential characteristics, firing frequency and spike-timing relative to evoking current stimuli. We then tested whether AUT1, a modulator of Kv3 K^+^ currents reverses the effects of sound exposure or TEA. Both loud sound exposure and TEA decreased the amplitude of action potential after-hyperpolarization, reduced the maximum firing frequency, and disrupted spike-timing. These treatments also increased post-synaptic voltage fluctuations at baseline. AUT1 applied in the presence of TEA or following acoustic over-exposure, did not affect the firing frequency, but enhanced action potential after-hyperpolarization, prevented the increased voltage fluctuations and restored spike-timing. Furthermore AUT1 prevented the occurrence of bursts. Our study shows that the effect on spike-timing is significantly correlated with the amplitude of the action potential after-hyperpolarization and the voltage fluctuations at baseline. In conclusion, modulation of putative Kv3 K^+^ currents may restore regular spike-timing of DCN fusiform cell firing following noise exposure, and could provide a means to restore deficits in temporal encoding observed during noise-induced tinnitus.

## Highlights

• Whole cell recordings were performed in dorsal cochlear nucleus fusiform cells.
• Spike-timing is dependent on the action potential after-hyperpolarization.
• Spike-timing is dependent on synaptic baseline voltage fluctuations.
• Inhibition of K^+^ currents using TEA or acoustic over-exposure disrupt spike-timing.
• AUT1, a Kv3.1/3.2 K^+^ current modulator, counteracts the disruptive effects on spike-timing.

## 1 Introduction

High-frequency action potential firing is essential for rapid information processing in the central nervous system, and in particular in the auditory system, which must encode complex auditory information with high fidelity (Carr, 1993, Joris and Yin, 2007, King et al., 2001).

Kv3.1 K^+^ channels mediate currents with a high activation threshold and fast activation and deactivation kinetics, allowing for rapid action potential repolarization and short inter-spike intervals (Erisir et al., 1999, Rudy et al., 1999, Rudy and McBain, 2001). Kv3.1 K^+^ current activation and deactivation properties explain why those currents are critical for permitting high frequency firing of neurons. In accordance with this observation, Kv3.1 K^+^ currents are expressed in neurones firing at high frequency such as in the spinal cord (Deuchars et al., 2001), cortex (Erisir et al., 1999), cerebellum (Joho and Hurlock, 2009) and auditory nuclei (Wang et al., 1998).

The dorsal cochlear nucleus (DCN) is an auditory brainstem structure playing a pivotal role in the integration of information from multiple sensory pathways (Wu and Martel, 2016) and in acoustic cues related to vertical sound source localization (May, 2000). DCN principal fusiform cells fire reliable and precise trains of action potentials in response to depolarizations (Ding et al., 1999, Hancock and Voigt, 2002a, Hancock and Voigt, 2002b, Manis, 1990, Oertel and Wu, 1989, Pilati et al., 2008). Our previous study has shown that acoustic over-exposure triggers hearing loss, and this correlated with profound changes in the firing pattern and frequency of DCN fusiform cells (Pilati et al., 2012). After acoustic over-exposure, a proportion (∼40%) of DCN fusiform cells display a distinct bursting firing pattern which has been associated with reduced Kv3.1 K^+^ currents, losing the ability to fire regularly and at high firing frequencies (Finlayson and Kaltenbach, 2009, Pilati et al., 2012). DCN fusiform cells also exhibit increased spontaneous firing rates (Brozoski et al., 2002, Dehmel et al., 2012, Kaltenbach et al., 2004) and increased cross-unit synchrony and bursting of spontaneous firing which correlate with behavioural measures of tinnitus (Finlayson and Kaltenbach, 2009, Kaltenbach et al., 1998, Wu and Martel, 2016). Despite evidence demonstrating firing frequency modulation and burst induction within the DCN (Finlayson and Kaltenbach, 2009, Pilati et al., 2012), the role of Kv3.1 K^+^ currents in DCN fusiform cell spike-timing remains unexplored. In this study we explore the effects of Kv3.1 K^+^ currents on the firing frequency and spike-timing of DCN fusiform cells. We used tetraethylammonium (TEA), a K^+^ channel blocker known to inhibit the Kv3 K^+^ currents at low concentrations (IC_50_ ∼0.3 mm) (Critz et al., 1993, Grissmer et al., 1994, Hernandez-Pineda et al., 1999, Johnston et al., 2010, Kanemasa et al., 1995) and acoustic over-exposure to trigger a down-regulation of high voltage-activated (Kv3 type) K^+^ currents (Pilati et al., 2012), to test the disruptive effects on spike timing. Firing precision of DCN fusiform cells was assessed using an analysis of the coefficient of variation (Pilati et al., 2012), and spike-time reliability was assessed by measuring the ability of the fusiform cell to fire consistently across repeated trials with the same current stimulus (Joris et al., 2006).

Until recently, the exploration of the role of Kv3 K^+^ channels in neurophysiology has been hampered by the absence of pharmacological tools. However, the compound (5R)-5-ethyl-3-(6-((4-methyl-3-(methyloxy)phenyl)oxy)-3-pyridinyl)-2,4-imidazolidinedione, (AUT1) has been shown to be a selective Kv3.1/3.2 K^+^ channel modulator (Rosato-Siri et al., 2015) increasing the open probability of Kv3 K^+^ channels, and shifting the voltage-dependence of activation of Kv3.1/3.2 K^+^ currents to more negative potentials (Brown et al., 2016, Rosato-Siri et al., 2015, Taskin et al., 2015). Here, we used AUT1 in the presence of a low concentration of TEA, or after acoustic over-exposure, to test whether positive modulation of Kv3.1 K^+^ currents could rescue impaired DCN fusiform cell firing precision and spike-time reliability.

Spike-timing depends upon various factors including the membrane time constant (Azouz and Gray, 2000), voltage-gated ion channels (Azouz and Gray, 2000, Fricker and Miles, 2000, Higgs and Spain, 2011, Jaeger and Bower, 1999), the coincident activation of pre-synaptic neurones (Diesmann et al., 1999, Gauck and Jaeger, 2003, Grande et al., 2004, Grothe and Sanes, 1994), and/or baseline spontaneous membrane voltage fluctuations (Dorval and White, 2005, Jaeger and Bower, 1999). We therefore tested whether modulation on spike-timing was dependent on baseline spontaneous membrane voltage fluctuations. As TEA (Erisir et al., 1999, Wang et al., 1998) and acoustic over-exposure (Pilati et al., 2012) reduced the amplitude of DCN fusiform cell action potential after-hyperpolarization, we also tested whether firing precision and spike-time reliability were dependent on the action potential after hyperpolarization.

Our study shows that Kv3.1 K^+^ currents control the spike-timing in DCN fusiform cells, via an effect on the action potential after-hyperpolarization and on spontaneous membrane voltage fluctuations. Our study further suggests Kv3.1 K^+^ currents act as a potential target to restore action potential fidelity following acoustic trauma.

## 2 Methods

### 2.1 Subjects

Male and female CBA mice aged between P15 and P18 were used. Experiments were carried out in accordance with the UK Animals (Scientific Procedures) Act of 1986 Home Office regulations and approved by the Home Office and Leicester University Ethical Committee (PIL 157DACE03, PPL 60/4351).

### 2.2 Dissection and slicing

Mice were culled by decapitation and the brainstem was dissected in an ice-cold low Na^+^ medium containing (in millimolar): KCl (2.5), glucose (10), NaH_2_PO4 (1.2), ascorbic acid (0.5), sucrose (250), NaHCO_3_ (26), CaCl_2_ (0.1) and MgCl_2_ (4), bubbled with 95% O_2_ and 5% CO_2_ to maintain a pH of 7.4. The brainstem was glued onto a chamber of a vibroslicer (Leica VT1000S). Coronal slices containing the dorsal cochlear nucleus were cut at a thickness of 200 μm in the ice-cold low Na^+^ medium described above. Slices were incubated at 35–37 °C for 1 h in an artificial cerebro-spinal fluid (ACSF) gassed with 95% O_2_ and 5% CO_2_ and containing (in millimolar): NaCl (125), KCl (2.5), glucose (10), NaH_2_PO_4_ (1.2), Na-pyruvate (2), myo-inositol (3), ascorbic acid (0.5), NaHCO_3_ (26), CaCl_2_ (2) and MgCl_2_ (1). Slices were then left in ACSF at room temperature (22–25 °C) for the remainder of the experiment. All chemicals were obtained from Sigma unless otherwise specified.

### 2.3 Acoustic over-exposure

Acoustic over-exposure was performed on P15 mice anaesthetised with an intraperitoneal injection of fentanyl (0.025 mg/kg), midazolam (2.5 mg/kg) and medetomidine (0.25 mg/kg). Mice were placed in a custom made open field sound-insulated chamber containing a 600 W High Power Horn Tweeter radiating evenly, frequency range 2–20 kHz (Maplin UK). Mice were exposed to a bilateral loud (110 dB SPL) single-tone frequency (14.8 kHz) sound for 2 h. Control animals were similarly anaesthetised at P15 but unexposed to loud sound.

### 2.4 Pre-pulse inhibition of the acoustic startle reflex

Hearing was assessed as the ability to respond to a lower intensity prepulse inhibiting the amplitude of the startle response. (Longenecker et al., 2014, Longenecker et al., 2016). The pre-pulse inhibition (PPI) of the acoustic startle reflex was assessed using a specific acoustic startle reflex hardware and software (Kinder Scientific, Poway, CA). A 110 dB SPL, 20 ms broadband noise stimulus served as the startle stimulus and was either presented on its own, or preceded by a prepulse applied for 50 ms, 100 ms before the startle stimulus, and with a 1 ms rise/fall time. Prepulses were presented as octave-based 12 or 24 KHz frequencies applied at 70 dB SPL. For each frequency, the mice were given 12 startle stimulus-alone trials, intermingled with 12 trials containing a prepulse. Each session started by two startle stimuli (with no prepulse) to allow acclimatization to the testing procedure. This was subsequently excluded from the analysis. A ratio was then calculated by dividing the amplitude of the startle response recorded following the prepulse by the amplitude of the startle response during a startle only trial. Mice were tested at P13 (prior to acoustic over exposure) and at P17 (following acoustic over-exposure) with ratio elevations considered as the behavioural evidence of hearing loss (Longenecker et al., 2014, Longenecker et al., 2016).

### 2.5 Whole-cell recordings

Whole cell recordings were conducted at P14-P18. When mice were anesthetised (section 2.3) or subject to acoustic over-exposure (section 2.4), whole cell recordings were performed at P17-P18. Coronal slices containing the dorsal cochlear nucleus were placed in a recording chamber mounted on the stage of an upright microscope (Axioskop; Carl Zeiss, Oberkochen, Germany), and perfused with ACSF at room temperature (22–25 °C) at a rate of 1 ml/min. Fusiform cells were identified by their morphology, passive properties, position and orientation within the fusiform layer (Pilati et al., 2008). Whole cell recordings were performed using a Digidata 1200 interface (Axon Instruments, Foster City, CA), a Multiclamp 700 A amplifier (Molecular Devices Inc. USA) and PClamp 9.2 software (Molecular Devices Inc. USA). Data were acquired with a sampling rate of 20 kHz and filtered at 4 kHz. Unless otherwise stated, cells were held at -80 mV. Liquid junction potential (calculated to be 1.6 mV) was not corrected for. Whole-cell recordings were carried out in ACSF at room temperature.

#### 2.5.1 Whole-cell current clamp recordings

Patch pipettes were made of borosilicate glass (4–7 MΩ tip resistance) contained (in millimolar): KCl (110), EGTA (0.2), HEPES (40), MgCl_2_ (1), CaCl_2_ (0.1), Na_2_phosphocreatine (5), l-arginine (1), pH 7.1–7.2 adjusted with KOH.

#### 2.5.2 Whole-cell voltage clamp recordings

The intracellular medium was (in millimolar): Kgluconate (116), EGTA (11), HEPES (40), MgCl_2_ (4), CaCl_2_ (0.45), Na_2_phosphocreatine (5), l-arginine (1), pH 7.1–7.2 adjusted with KOH when recordings were performed in paired conditions (Fig. 2A). The composition of the intracellular medium matched the intracellular medium used for current clamp recordings when recordings were performed in the unpaired condition (Fig. 2B). Tetrodotoxin (1  μM) (Abcam) was present in the ACSF to block sodium currents. Series resistance (<15 MΩ) was compensated by 70%. Holding potential was −60 mV and voltage steps from −70 mV to +40 mV (in 10 mV increments) were preceded by a 1 s pre-pulse to −30 mV to partially inactivate low-voltage activated potassium currents (Brew and Forsythe, 1995).

**Fig. 1:**
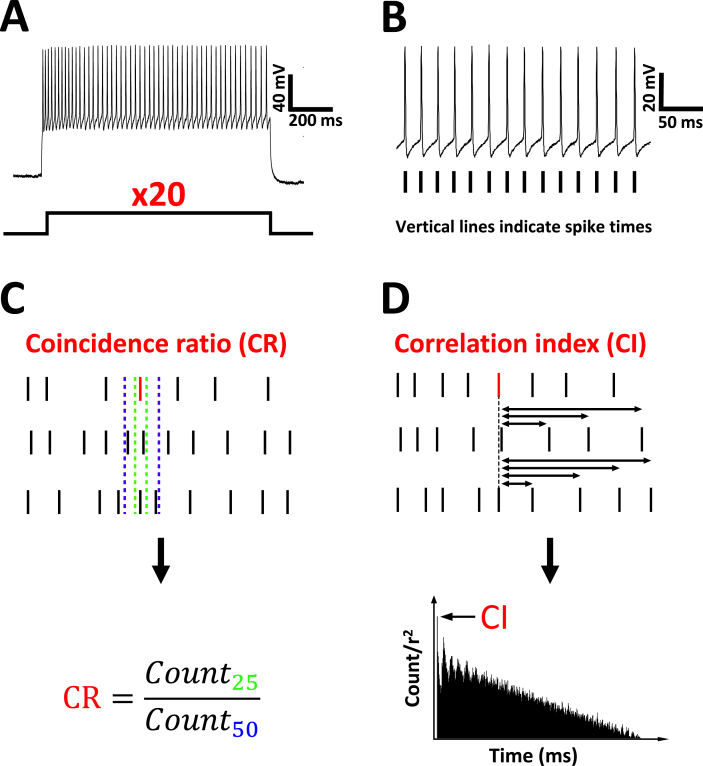
**The coincidence ratio and correlation index: two metrics used to quantify spike-time precision.** (**A**) Action potentials were elicited by delivering 20 identical current injections of 500 ms duration. Current amplitude was chosen to elicit action potentials at a firing frequency of 25–75 Hz. (**B**) Action potential peak times were obtained from each spike train and the coincidence of spikes occurring at the same time was measured using the coincidence ratio (**C**) or shuffled autocorrelation (**D**) technique. (**C**) The coincidence ratio (CR) technique consisted of counting spikes across each train, within a small and a large window set at either 25% (green) or 50% (blue) respectively, at both sides of the selected spike (red). A measure of spike-time precision was determined by dividing the 25% count (count_25_) by the 50% count (count_50_) (**C**, bottom). (**D**) The shuffled autocorrelation technique consisted of obtaining forward intervals for each spike (**D**, red). Forward interval counts were subsequently normalised to the mean firing frequency^2^ (r^2^) and plotted in a histogram (**D**, bottom). Spike-time precision was taken as the height of the first peak (**D**, arrow) and termed the correlation index (CI). (For interpretation of the references to colour in this figure legend, the reader is referred to the Web version of this article.)

**Fig. 2:**
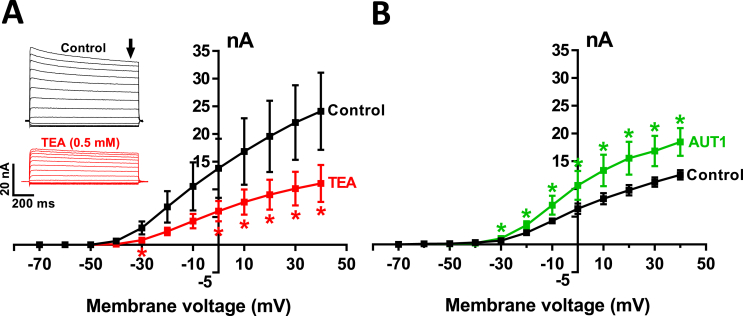
**Effects of TEA and AUT1 on outward currents in dorsal cochlear nucleus fusiform cells.** (**A**) Mean current-voltage relationships in the control condition (black) and after TEA perfusion (red). Holding potential was −60 mV and 1 s pre-pulse to −30 mV preceded voltage steps from −70 mV to +40 mV (10 mV increments). *P < 0.05; n = 4, one-tailed paired t-tests. Inset shows the current traces from a single cell, in absence (control) and presence of 0.5 mM TEA. The arrow points to the time of measurement. (**B**) Mean current-voltage relationships for outward currents in the control condition (black) and in presence of 10 μM AUT1 (green). Data were obtained in unpaired conditions during which slices were pre-incubated with AUT1 for a minimum of 1 h *P < 0.05; one-tailed unpaired t-tests, control n = 6; AUT1 n = 6. All data are with leak subtraction and presented as mean ± SEM. (For interpretation of the references to colour in this figure legend, the reader is referred to the Web version of this article.)

### 2.6 Compounds and pharmacological testing

Tetraethylammonium (TEA) chloride (Sigma-Aldrich) was used to inhibit K^+^ currents including Kv1, Kv3, Kv7 and BK Ca^2+^ activated K^+^ currents (Brew and Forsythe, 1995, Johnston et al., 2010). Tetraethylammonium (TEA) was used at a concentration of 0.5 mM, shown to partially block Kv3 K^+^ currents (Erisir et al., 1999, Hernandez-Pineda et al., 1999, Rosato-Siri et al., 2015). AUT1 (Autifony Therapeutics Ltd) was used as a Kv3.1/3.2 K^+^ channel modulator as previously described (Brown et al., 2016, Rosato-Siri et al., 2015, Taskin et al., 2015). Stock solutions of TEA were made in water and stored at 4 °C. Stock solutions of AUT1 were made in DMSO and diluted in ACSF to obtain a final AUT1 concentration of 10 μM for experiments involving acoustic over-exposure or 30 μM AUT1 in presence of TEA (maximal DMSO final concentration was 0.3%). Stock solutions of AUT1 were stored at −20 °C. Unless otherwise stated, slices were incubated with the desired final concentration of DMSO, TEA and/or AUT1 at least 1 h prior to recording (unpaired conditions), to ensure full equilibration of the compounds (Rosato-Siri et al., 2015). DMSO concentrations were matched for all recording solutions. No time-related incubation effects were observed on the measured parameters.

### 2.7 Data analysis

#### 2.7.1 Spike detection

Action potentials were visually selected in the first instance and then detected automatically using Clampfit (PClamp version 9.2, Molecular Devices). Automatic selection was subsequently manually checked.

#### 2.7.2 Single action potentials

Single action potentials were elicited at threshold potentials (usually with current injections of less than 400 pA). The action potential peak value was obtained at the maximal point of the over-shoot, whereas the action potential amplitude was measured as the difference between the action potential threshold voltage and its peak. The action potential half-width was measured as the time corresponding to the depolarization and the repolarization phases at half height. Action potential 10–90% rise time and 90-10% decay time were measured from the threshold to the peak and the peak to the lowest point of the after-hyperpolarization respectively. After-hyperpolarization was taken as the negative area (mV.ms^−1^) below the baseline following the action potential peak and divided into 5 ms bins, allowing the analysis of fast and slow components of the after-hyperpolarization.

#### 2.7.3 Firing properties

Fusiform cells were injected with 1 s current steps (varying from 50 pA to 1500 pA, with 50 pA increments). Firing frequency was analysed on action potentials elicited at −40 mV. The firing frequency was measured as the number of action potentials divided by the difference in time between the first and last action potential occurring in the step. The spike-rate adaptation was calculated as a spike frequency ratio, on a 1 s pulse exceeding 0.7 nA, using 1/(F_L200 ms_/F_F200ms_), where F_L200 ms_ and F_F200ms_ correspond to the firing frequencies recorded during the last and the first 200 ms period of the step respectively. The precision of the neural response was quantified by calculating the inter-spike interval (ISI) variation using a coefficient of variation (CV) calculated as (SD_ISI_/mean_ISI_) where SD_ISI_ and mean_ISI_ are the mean and the standard deviation of the ISIs respectively. A burst was typically defined as one or more action potentials occurring in close succession to one another relative to the average ISI from the same constant current stimulus (Finlayson and Kaltenbach, 2009). The reliability of the neural response was quantified as the ability of fusiform cells to fire action potentials over repeated trials, measured using the method of the coincidence ratio (CR). Twenty identical current steps (500 ms sweeps) were injected into a cell (Fig. 1A) and action potential peak times were obtained (Fig. 1B). Current amplitude was chosen to elicit action potentials firing at a frequency of 25–75 Hz (Manis, 1990, Pilati et al., 2012). Time stamps across action potential trains were set within a small (Fig. 1C, green lines) and a large (Fig. 1C, blue lines) window, corresponding to 25% and 50% of the average ISI respectively. Action potentials were then counted within those 2 windows, and a coincidence ratio was obtained by dividing the 25% count by the 50% count (Count_25%_/Count_50%_). For each cell, a coincidence ratio was obtained by averaging the coincidence ratio for all 20 sweeps. To confirm the results from the coincidence ratio, spike-time reliability was also measured using the previously published shuffled autocorrelation technique (Joris et al., 2006, Street and Manis, 2007). For each spike of each train the forward intervals were collected (Fig. 1D, top) and subsequently plotted in a histogram normalised by the square of the mean firing frequency (r^2^) (Fig. 1D, bottom). The height of the first peak of this autocorrelation histogram (Fig. 1D, arrow), termed correlation index (CI), is taken as a measure of spike-time reliability.

#### 2.7.4 Membrane potential fluctuations

Membrane potential fluctuations were recorded for a 3 s period at a holding potential of −80 mV. Voltage fluctuations consisted of a mixture of excitatory and inhibitory post-synaptic potentials observed as outward deflections. Voltage fluctuations were blocked by 1 mM kynurenic acid (Sigma-Aldrich), 10 μM NBQX (2,3-dihydroxy-6-nitro-7-sulfamoyl-benzo[f]quinoxaline-2,3-dione, Ascent Scientific), 10 μM gabazine (Abcam) and 10 μM strychnine (Abcam) (n = 4, data not shown). Membrane potential fluctuations were quantified by obtaining the baseline to peak amplitude for each time point during a 3 s recording. The baseline was taken as the steady state voltage (absence of voltage fluctuations) at −80 mV and was visually determined. Cumulative distribution plots of the voltage amplitudes were then constructed and differences were assessed using Kolmogorov-Smirnov tests.

### 2.8 Statistics

Data were analysed using Clampfit 9.2 (Molecular Devices) and analysed using custom written routines in MATLAB (Version R2016a) and Graphpad Prism (version 7). Data distributions were tested for normality using a Shapiro-Wilk test. A one way analysis of variance (ANOVA) with post-hoc Tukey test was used to test for differences between groups when the data were normally distributed, and an ANOVA on Ranks (Kruskal-Wallis) test with follow-up Mann-Whitney (two-sided) test was used when data were not normally distributed. To avoid type 1 errors when using the Mann-Whitney tests, P-values were adjusted according to Bonferonni correction. Subthreshold voltage amplitudes were plotted in a cumulative distribution plot and differences between conditions were determined using a Kolmogorov-Smirnov test. Individual data on coefficient of variation, action potential after-hyperpolarization, subthreshold voltage fluctuations and the coincidence ratios were found to be non-parametric and were therefore tested for correlations using Spearman's Rho tests. One-sided paired t-tests were used when testing a decrease or an increase of the K^+^ current amplitude in presence of TEA or AUT1 respectively. Unless otherwise specified, data are presented as mean ± SEM. Results are considered statistically significant when P < 0.05.

## 3 Results

### 3.1 Effects of TEA and AUT1 on K^+^ currents in DCN fusiform cells

Whole cell voltage clamp recordings were performed on DCN fusiform cells, which were held at −60 mV. Stepping to voltages above −30 mV evoked large non-inactivating outward K^+^ currents (Fig. 2A, current at + 0 mV = 16.54 ± 10 nA, n = 4) which were reduced by approximately 50% by the perfusion of 0.5 mM TEA (Fig. 2A , 8.1 ± 3.6 nA, n = 4, P = 0.04, paired *t*-test). Slices were pre-incubated with AUT1 (10 μM) for 1 h (Rosato-Siri et al., 2015). This resulted in a 65% increase in the amplitude of the K^+^ current at 0 mV (Fig. 2B, Control: 6.4 ± 2.3 nA, n = 6; AUT1: 10.6 ± 6.3 nA, n = 6, P = 0.02, one-sided Mann-Whitney test).

### 3.2 AUT counteracts the effect TEA on action potential after-hyperpolarization

**Fig. 3:**
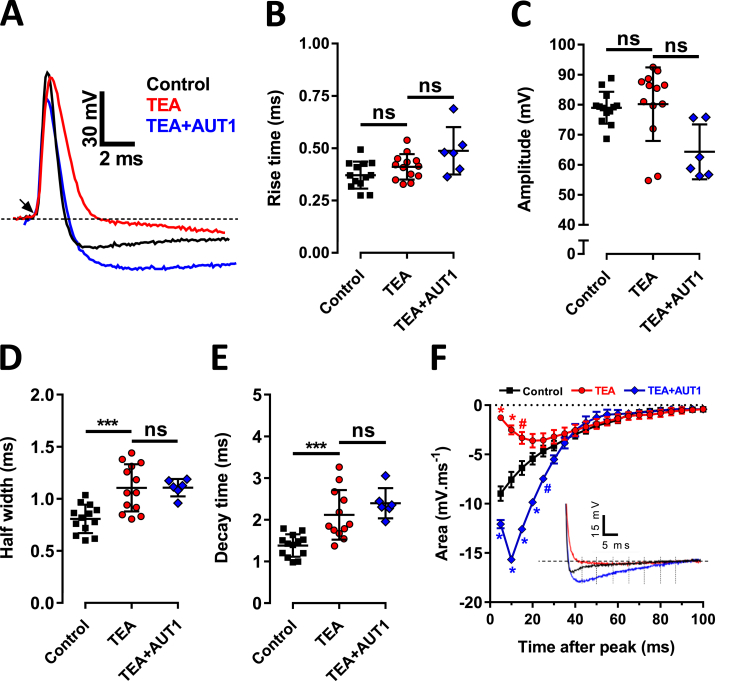
**AUT1 restores action potential after-hyperpolarization following K**^**+**^**current inhibition.** (**A**) Example of single action potentials in response to low (<400 pA) current injections, in control (black), TEA (red) and TEA + AUT1 (blue, AUT1) conditions. Action potentials are aligned by their threshold (arrow). (**B**–**E**) Plot of action potential rise time (**B**), amplitude (**C**), half-width (**D**) and decay time (**E**), in control (n = 13, black), TEA (n = 13, red) and TEA + AUT1 (n = 6, blue, AUT1) conditions. TEA had no effect on action potential rise time or amplitude but did cause a significant increase in half-width (P = 0.0004) and decay time (P = 0.0008). AUT1 when in presence of TEA had no effect on rise time (**B**) or amplitude (**C**), and did not restore the half-width (**D**) or decay time (**E**). (**F**) Measures of after-hyperpolarization area were divided into 5 ms bins. Insert shows an expanded view of typical action potential after-hyperpolarizations for control (black), TEA (red) and TEA + AUT1 (blue, AUT1). The horizontal dashed line in the insert indicates the baseline voltage in response to a low current injection (<400 pA) from a holding potential of −80 mV. The vertical dashed lines in the insert indicate the 5 ms bins (see methods). TEA caused a significant decrease in the size of the after-hyperpolarization within the first 15 ms from the action potential peak, as indicated by a reduction in after-hyperpolarization area, and AUT1 could prevent this. (**A**) Holding potential = −80 mV. (**B**–**E**) Error bars = SD. (**F**) Error bars = SEM. (**D**,**E**) ***P < 0.001. (**F**) #P < 0.001; *P < 0.0001. (For interpretation of the references to colour in this figure legend, the reader is referred to the Web version of this article.)

Whole cell current clamp recordings were obtained from DCN fusiform cells. Resting membrane potentials measured in presence of TEA (0.5 mM), AUT1 (10 μM) were similar to those recorded in control conditions (control: −50.4 ± 1.2 mV (n = 10); AUT1: −48.3 ± 1.1 mV (n = 10), TEA: −49.8 ± 0.9 (n = 11), ANOVA; P = 0.37). Single action potentials were obtained in response to low intensity currents (<400 pA) and were evoked at a threshold potential of −43 ± 3.9 mV. Action potentials were recorded in control conditions (n = 13) and were characterised by a rise time of 0.37 ± 0.06 ms (Fig. 3A and B), an amplitude of 79 ± 5 mV (Fig. 3A,C), a peak of 36 ± 5 mV, a half-width of 0.81 ± 0.1 ms (Fig. 3A,D) and a decay time of 1.4 ± 0.3 ms (Fig. 3A,E). Action potentials in control conditions were also characterised by an after-hyperpolarization of −9.0 ± 0.73 mV ms^−1^ (measured at 5 ms after the peak, Fig. 3F). TEA (n = 13) did not affect the action potential threshold (−46.1 ± 3.9 mV, ANOVA and Tukey post-hoc: P = 0.1), rise time (Fig. 3A,B, 0.41 ± 0.06 ms, ANOVA and Tukey post-hoc: P = 0.37) or amplitude (Fig. 3A,C, 80 ± 12 mV, Mann-Whitney: U = 56, P = 0.3), but increased the action potential half-width (Fig. 3A,D, 1.1 ± 0.2 ms, ANOVA and Tukey post-hoc: P = 0.0004) and decay time (Fig. 3A,E, 2.1 ± 0.6 ms, ANOVA and Tukey post-hoc: P = 0.0008). TEA also reduced the amplitude of the after-hyperpolarization (Fig. 3F, −1.2 ± 0.31 mV ms^−1^, measured 5 ms after the peak, ANOVA and Tukey post-hoc test: P < 0.0001). We next tested the ability of AUT1 to counteract the effects of TEA. Slices (n = 6) were incubated in TEA and AUT1 prior to recording. In the presence of both TEA and AUT1, the action potential half-width (1.1 ± 0.08 ms n = 6, Fig. 3A,D) and decay time (2.4 ± 0.4 ms, n = 6, Fig. 3A,E) were similar to TEA alone (ANOVA and Tukey post-hoc test: P = 0.99 and P = 0.4, respectively). Although action potential thresholds were more depolarized in the presence of TEA and AUT1 (−35.27 ± 2.4 mV, n = 6) compared to TEA alone (ANOVA and Tukey post-hoc: P < 0.0001), this did not significantly affect the amplitude of the action potentials which remained similar between the two conditions (Fig. 3A,C, amplitude in TEA: 80 ± 12 mV, n = 13; TEA + AUT1: 64.3 ± 9.1 mV, n = 6, Mann-Whitney: U = 14, P = 0.057). Action potential peak values were also similar between TEA and TEA + AUT1 conditions (TEA: 34.1 ± 14 mV, n = 13; TEA + AUT1 29 ± 7.8 mV, n = 6, Kruskal-Wallis test and Mann-Whitney post-hoc: P = 0.16). The amplitude of the after-hyperpolarization was restored in the presence of TEA and AUT1 compared to TEA conditions and even increased by ∼35% compared to control conditions (Fig. 3F, after-hyperpolarization: −12.1 ± 0.42 mV ms^−1^ at 5 ms following the peak, n = 6, ANOVA and Tukey post-hoc test: P < 0.0001).

### 3.3 AUT1 does not prevent the effects of TEA on firing frequency and spike-rate adaptation

**Fig. 4:**
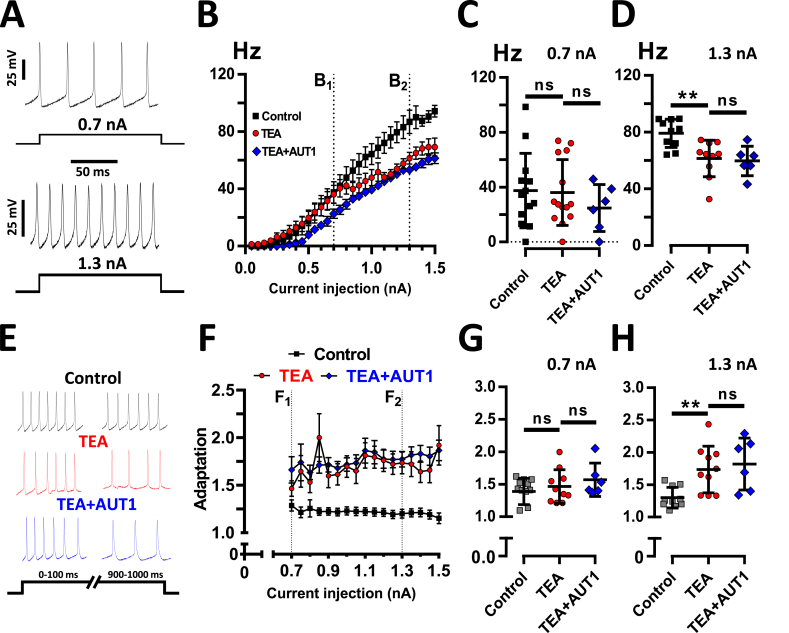
**Absence of effect of AUT1 on firing frequency and spike rate adaptation following K**^**+**^**current inhibition.** (**A**) Typical examples of action potential firing in response to a current injection of 0.7 nA (top) and 1.3 nA (bottom) in the control condition. (**B**) Frequency-current intensity relationships obtained in control (n = 13), TEA (n = 14) and TEA + AUT1 (n = 6, AUT1). (**C**) Plot of firing frequencies measured at 0.7 nA (dashed line, **B1**). Neither TEA nor AUT1 in presence of TEA had an effect. (**D**) Plot of firing frequencies measured at 1.3 nA (dashed line, **B2**). TEA reduced the firing frequency (P = 0.0043) and AUT1 failed to prevent this (P = 0.94). (**E**) Example traces showing firing rates during the first and the last 100 ms of a 1000 ms depolarizing pulse, in the different conditions. (**F**) Plot of spike train adaptation across increasing current injections. Adaptation was calculated using 1/(FL_200 ms_/FF_200ms_), where FL_200 ms_ and FF200ms correspond to the firing frequencies recorded during the last and the first 200 ms period of a 1000 ms step respectively. (**G**) Plot of spike train adaptation measured at 0.7 nA (dashed line, **F1**). Neither TEA nor AUT1 in presence of TEA had an effect. (**H**) Plot of spike train adaptation measured at 1.3 nA (dashed line, **F2**). TEA increased spike train adaptation (P = 0.0123) and AUT1 failed to prevent this (P = 0.85). (**B**,**F**) Error bars = SEM. (**C**-**D**, **G**-**H**) Error bars = SD. (**D**,**H**) **P < 0.01.

Similar to previous studies, fusiform cells fired action potentials with an increasing frequency in response to increasing current injection, reaching a maximal frequency of _∼_100 Hz in response to a 1.3–1.5 nA current injections (Fig. 3A and B). The firing frequency was decreased in presence of TEA in response to high current injections (at 1.3 nA: control: 79.2 ± 9.9 Hz, n = 10; TEA: 61.5 ± 12.9 Hz, n = 10, ANOVA and Tukey post-hoc: P = 0.0043) (Fig. 4B,D). The firing frequency of _∼_40 Hz in response to low (0.7 nA) current injection was unaffected by the presence of TEA (Fig. 4B and C, control: 37.5 ± 27 Hz, n = 13; TEA: 36.1 ± 24 Hz, n = 14, ANOVA and Tukey post-hoc: P = 0.98), consistent with TEA inhibiting a proportion of high voltage activated K^+^ currents in DCN fusiform cells (Pilati et al., 2012). TEA also increased the spike-rate adaptation, observed at high (1.3 nA) current injections (Fig. 4E,F,H, spike frequency ratio: 1.3 ± 0.16, n = 10; TEA: 1.74 ± 0.36, n = 10, ANOVA and Tukey post-hoc: P = 0.0123) whereas it left the spike-rate adaptation at low (0.7 nA) current injections unaffected (Fig. 4E,F,G, spike frequency ratio: 1.39 ± 0.2, n = 12; TEA: 1.47 ± 0.26, n = 10, Mann-Whitney: U = 55, P = 0.77). The firing frequency measured for high (1.3 nA) current injections was 59.7 ± 10.5 Hz (n = 6) in the presence of TEA and AUT1 (Fig. 4B,D) compared to 61.5 ± 12.9 Hz in the presence of TEA (n = 10, ANOVA and Tukey post-hoc test: P = 0.94) and 79.2 ± 9.9 Hz in control (n = 10, ANOVA and Tukey post-hoc test: P = 0.008). The spike-rate adaptation was 1.82 ± 0.4 (n = 6) in the presence of TEA and AUT1 (Fig. 4E,F,H) compared to 1.74 ± 0.36 in the presence of TEA (n = 10, ANOVA and Tukey post-hoc test: P = 0.85) and 1.3 ± 0.16 in control (n = 10, ANOVA and Tukey post-hoc test: P = 0.0095).

### 3.4 AUT1 counteracts the effects of TEA on spike time precision

**Fig. 5:**
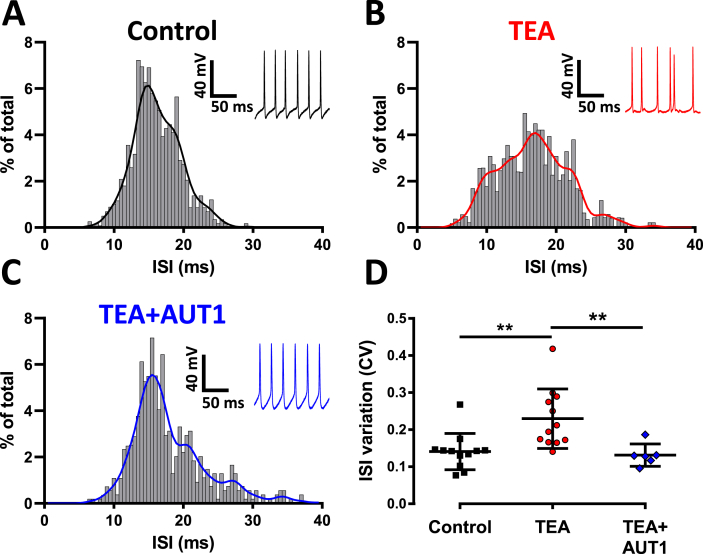
**AUT1 restores inter-spike interval regularity following deficits caused by K**^**+**^**current inhibition**. Histograms show the inter-spike interval (ISI) distributions for control (**A**), TEA (**B**) and TEA + AUT1 (AUT1) (**C**). (**A**–**C**) Data were collected from action potential trains firing between 25 and 75 Hz and are represented as percentages of 11719 (**A**), 9496 (**B**) and 10908 (**C**) events. Insets show example traces. (**D**) Plot of the ISI variation for each condition measured using the coefficient of variation. TEA increased the ISI variation (P = 0.002) and AUT1 could prevent this (P = 0.02). Control (n = 12), TEA (n = 12), TEA + AUT1 (n = 6, AUT1). (**D**) Error bars = SD. *P < 0.05, **P < 0.01.

Fusiform cells recorded in control conditions fired regular action potentials (Fig. 5A, insert), as shown by the narrow distribution of inter-spike intervals (Fig. 5A) and the related coefficient of variation of 0.14 ± 0.05 (Fig. 5D, n = 12, black). By contrast, irregular (burst-like) inter-spike intervals were observed in presence of TEA (Fig. 5B, insert). This was reflected in their wider distribution (Fig. 5B) and an increased coefficient variation (Fig. 5D, control: 0.14 ± 0.05, n = 12; TEA: 0.23 ± 0.08, n = 12, Mann-Whitney test: U = 18, P = 0.002). Burst-like firing was absent when recordings were performed in presence of TEA and AUT1 and inter-spike intervals were regular in those conditions (Fig. 5C insert), as illustrated by the narrow inter-spike interval distribution (Fig. 5C). Fig. 5D shows the decreased inter-spike interval coefficient of variation in presence of AUT1 compared to TEA (TEA: 0.23 ± 0.08, n = 12; TEA + AUT1: 0.13 ± 0.03, n = 6, Mann-Whitney test: U = 47, P = 0.02).

### 3.5 AUT1 counteracts the effects of TEA on spike-time reliability

**Fig. 6:**
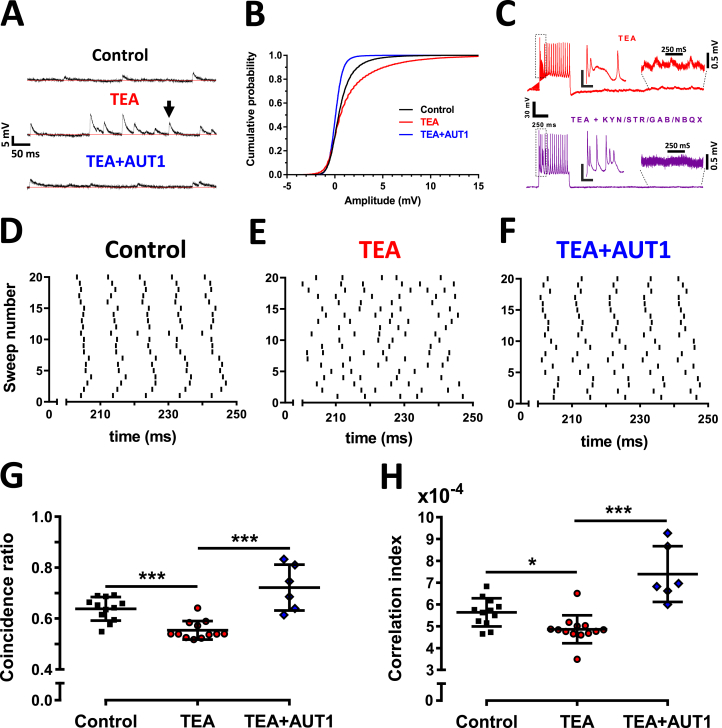
**AUT1 prevents increases in baseline voltage fluctuation and restores spike-time precision following K**^**+**^**current inhibition.** (**A**) Example traces showing membrane voltage in control (black), TEA (red) and TEA + AUT1 (blue, AUT1) conditions. (**B**) Cumulative probability distribution plots for the membrane voltage amplitude (voltage amplitude was taken relative to the baseline which was visually determined and is illustrated by the red lines in **A)**. Data used for the cumulative distributions consisted of 405262 (black, control), 537914 (red, TEA) and 254696 (blue, TEA + AUT1) individual baseline points. TEA produced a significant rightward shift in the cumulative distribution towards larger voltage amplitudes (Fig. 5**B**, Control: black, n = 13; TEA: red, n = 14; Kolmogorov-Smirnov test: P = <0.0001), as indicated by the increase in the amplitude of baseline voltage fluctuation (**A**, middle panel, arrow), and this was prevented by AUT1 (Fig. 5**A,B**, TEA: red, n = 14; TEA + AUT1: blue, n = 6; Kolmogorov-Smirnov test: P = <0.0001). (**C**) Example traces showing action potential trains and a section of baseline voltage proceeding in the TEA (red, top panel) condition and after perfusion of TEA plus blockers of synaptic transmission (purple, lower panel, NBQX 10 μM, kynurenic acid 1 mM, strychnine 10 μM and gabazine 10 μM). Blockers of synaptic transmission decreased the amplitude of baseline voltage fluctuation (right insets) but failed to prevent the accompanying bursts induced by presence of TEA (middle insets). Middle insets comprising of 3–7 action potentials correspond to areas of the action potential trains encompassed by dashed boxes. Scale marks related to expanded traces are 25 ms and 25 mV. (**D**,**E**,**F**) Fusiform cells were held at 80 mV and a repeated current stimulus (500 ms) was injected every 6 s. Raster plots show typical examples of spike-timing across 20 trials in single cells recorded in control (**D**), TEA (**E**), and TEA + AUT1 (**F**, right, AUT1). (**G**) Plot showing the effects of AUT1 and TEA on the coincidence ratio (see methods). TEA decreased the spike coincidence ratio (P = 0.0002) and AUT1 could prevent this (P = 0.0008). (**H**) Plot showing similar effects on the correlation index (see methods). (**G**–**H**) Error bars = SD. ns = non-significant. Control (n = 12), TEA (n = 12), TEA + AUT1 (n = 6, AUT1). **P < 0.01, ***P < 0.001. (For interpretation of the references to colour in this figure legend, the reader is referred to the Web version of this article.)

The dependence of action potential timing on subthreshold membrane fluctuation has previously been measured using a protocol consisting of repeated presentations of a low-pass filtered (frequency cut-off ∼ 1 kHz) white noise stimulus, and measuring how closely action potential initiation follows local maxima in the injected noise (Mainen and Sejnowski, 1995). Considering that fusiform cells follow local maxima in injected noise with high precision (Street and Manis, 2007), we conjectured that an increase (or decrease) in membrane potential fluctuation due to K^+^ channel modulation will regulate the ability for fusiform cells to fire action potentials in a reliable manner across repeated trials of a DC current injection. We assessed the effects of K^+^ current modulation on the regulation of membrane potential fluctuations, and any subsequent effects that this may have on spike-timing. Membrane voltage fluctuations in control conditions (0.73 ± 1.48 mV, n = 13, Fig. 6A top) were increased in the presence of TEA as shown by the rightwards shift of the cumulative probability distribution of the baseline voltage (see methods) in the presence of TEA (Fig. 6B, Control: black, n = 13; TEA: red, n = 14; Kolmogorov-Smirnov test: P = <0.0001). Perfusion of NBQX (10 μM), kynurenic acid (1 mM), strychnine (10 μM) and gabazine (10 μM) abolished baseline membrane potential fluctuations under control conditions (n = 4, not shown) and in presence of TEA (n = 4, Fig. 6C, lower panel) demonstrating that they are principally due to the background synaptic activity rather than other sources of membrane fluctuation such as channel noise (Yarom and Hounsgaard, 2011). Furthermore, the increase in membrane voltage fluctuations was not due to an increase in membrane resistance in presence of TEA, as this remained stable compared to the control condition (Control: 63.8 ± 25.54 MΩ, n = 13; TEA: 62.7 ± 29.7 MΩ, n = 13, Mann Whitney test: P = 0.75). Fig. 6C also shows that blocking synaptic transmission specifically affects membrane potential fluctuations in the presence of TEA, while leaving burst firing unaffected. Fusiform cell action potentials were studied in response to twenty identical current injections to obtain a measure of spike-time reliability. In control conditions, action potentials fired reliably across trials as visualised in the raster plot of spike times in Fig. 6D. TEA disrupted the ability of fusiform cells to fire reliably across trials (Fig. 6E), resulting in a decreased coincidence ratio (Fig. 6G, control: 0.64 ± 0.05, n = 12; TEA: 0.55 ± 0.04, n = 12, Mann-Whitney test: U = 10, P = 0.0002) and a decreased correlation index (Fig. 6H, control 5.6 × 10^−6^ ± 0.6 × 10^−6^, n = 12; TEA: 4.9 × 10^−6^ ± 0.64 × 10^−6^, n = 13, Mann-Whitney test: U = 29.5, P = 0.014). By contrast reliable firing across trials was observed when AUT1 was in presence of TEA (Fig. 6F) and this was demonstrated by an increased coincidence ratio and correlation index when compared to TEA (Fig. 6G, coincidence ratios: TEA: 0.55 ± 0.04, n = 12; TEA + AUT1: 0.72 ± 0.09, n = 6, Mann-Whitney test: U = 2, P = 0.0008; Fig. 6H, correlation index:TEA: 4.9 × 10^−6^ ± 0.64 × 10^−6^, n = 13; TEA + AUT1: 7.4 × 10^−6^ ± 1.3 × 10^−6^, n = 6, Mann-Whitney test: U = 1, P = 0.0002). Furthermore, when AUT1 was in presence of TEA, there was a leftwards shift in the cumulative probability distribution of baseline voltage relative to the TEA condition (Fig. 6B, TEA: red, n = 14; TEA + AUT1: blue, n = 6; Kolmogorov-Smirnov test: P = <0.0001), illustrating a decrease in membrane potential fluctuation in presence of TEA.

### 3.6 AUT1 counteracts the inhibitory effects of acoustic over-exposure on the action potential after-hyperpolarization

We next investigated the effects of AUT1 on the properties of the action potential and firing properties affected by acoustic over-exposure (Pilati et al., 2012). Hearing loss following acoustic over-exposure was assessed from the inability of animals to respond to a 70 dB SPL prepulse to inhibit the amplitude of the startle response (Longenecker et al., 2016). PPI ratios (see methods) were 0.37 ± 0.05 and 0.34 ± 0.06 for 12 and 24 KHz respectively prior to anaesthesia and remained similar post anaesthesia (sham procedure) (12 kHz: 0.41 ± 0.09, paired *t*-test: P = 0.21; 24 kHz: 0.32 ± 0.07, paired *t*-test: P = 0.64, n = 4). Although PPI ratios were similar before and after acoustic-over exposure for 12 KHz (0.42 ± 0.05 and 0.04 ± 0.06 respectively, paired *t*-test: P = 0.49, n = 5), they were increased (from 0.29 ± 0.11 to 0.65 ± 0.05, paired *t*-test: P < 0.001, n = 5) for 24 KHz showing a specific elevation of hearing threshold for a frequency above the frequency of acoustic over exposure (Pilati et al., 2012). Acoustic over–exposure did not affect the amplitudes of the startle responses when the startle stimulus was presented on its own (0.08 ± 0.018 N and 0.1 ± 0.044 N before and after acoustic over-exposure respectively, paired *t*-test: P = 0.3, n = 5), showing that the effect was specific to lower intensity prepulses.

**Fig. 7:**
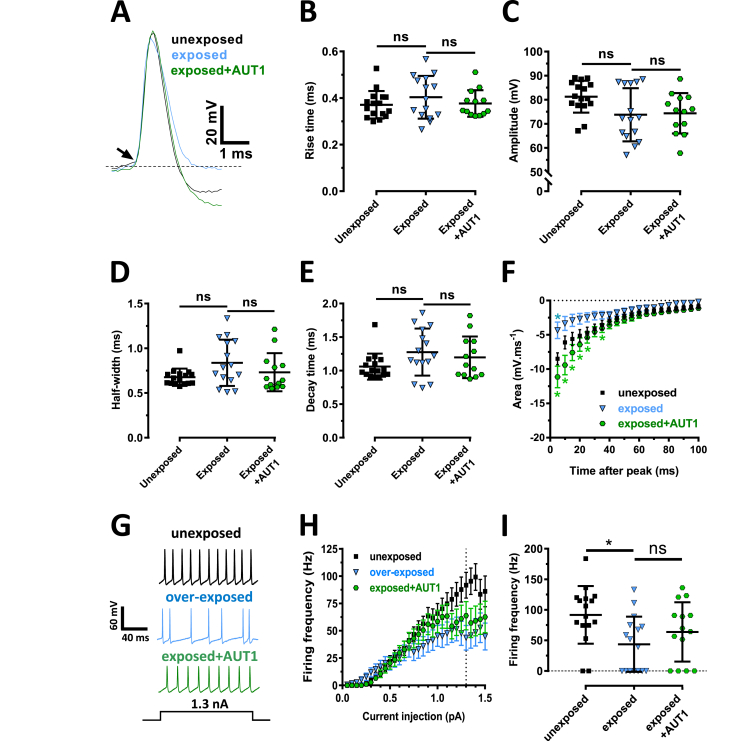
**Effect of positive K**^**+**^**current modulation on single action potential properties and firing frequency following acoustic over-exposure**. (**A**) Example action potentials in response to low (<400 pA) current injections, in the unexposed (black), exposed (blue) and exposed + AUT1 (green, AUT1) conditions. Action potentials are aligned by their threshold (arrow). (**B**–**E**) Plot of action potential rise time (**B**), amplitude (**C**), half-width (**D**) and decay time (**E**), in the unexposed (n = 16, black), exposed (n = 15, blue) and exposed + AUT1 (n = 13, green, AUT1) conditions. Neither acoustic over-exposure nor AUT1 had an effect on properties of single action potentials. (**F**) Acoustic over-exposure caused a reduction in the after-hyperpolarization area and this effect was prevented by AUT1. (**G**) Examples of action potential spike trains recorded in fusiform cells in response to 1.3 nA current injections. Traces originate from an unexposed mouse (top), a mouse previously subjected to acoustic over-exposure (middle), and a mouse previously subjected to acoustic over-exposure following the addition of AUT1 (bottom). (**H**) Frequency-current intensity relationships obtained in the unexposed (black, n = 16), exposed (blue, n = 16), and exposed + AUT1 (green, n = 14, AUT1) conditions. (**I**) Plot of firing frequency at 1.3 nA (dashed line in **H**). Firing frequency was taken as zero if a cell failed to fire action potentials. There was a significant decrease in firing frequency in mice that had previously been exposed to loud sound (P = 0.009) and AUT1 failed to prevent this effect (P = 0.48). (**B**-**E**,**I**) Error bars = SD. (**F**,**H**) Error bars = SEM. *P < 0.05. (For interpretation of the references to colour in this figure legend, the reader is referred to the Web version of this article.)

Resting membrane potentials of DCN FCs, measured following acoustic over-exposure, were similar to those recorded following sham exposure (unexposed: −62.11 ± 2.7 mV, n = 9; exposed: −61.7 ± 3.7 mV, n = 10, unpaired *t*-test: P = 0.79). Similarly to TEA, the after-hyperpolarization was decreased following acoustic over-exposure (measured in the first 5 ms following its peak, Fig. 7F, unexposed: −8.5 ± 0.88 mV ms^−1^, n = 16; exposed: −4.3 ± 1.2 mV ms^−1^, n = 15, Mann-Whitney test: U = 59.5, P = 0.031). Similarly to its effects in the presence of TEA, AUT1 counteracted the effects of acoustic over-exposure on the action potential after-hyperpolarization (Fig. 7F, exposed, after-hyperpolarization at 5 ms: 4.3 ± 1.2 mV ms^−1^, n = 15; exposed + AUT1, after-hyperpolarization at 5 ms: −11.13 ± 1.6 mV ms^−1^, n = 13, Mann-Whitney test: U = 43, P = 0.022). Effects of acoustic over-exposure were also studied on the properties of the single action potentials (Fig. 7A). Exposure to loud sound did not affect the action potential rise time (Fig. 7B, unexposed: 0.37 ± 0.06 ms, n = 16; exposed: 0.4 ± 0.09 ms, n = 15, Mann-Whitney test: U = 100, P = 0.9), amplitude (Fig. 7C, unexposed: 81.3 ± 6.6 mV, n = 16; exposed: 73.8 ± 11 mV, n = 15, Tukey post-hoc test: P = 0.06), half-width (Fig. 7D, unexposed: 0.68 ± 0.1 ms, n = 16; exposed: 0.84 ± 0.3 ms, n = 15, Mann-Whitney test: U = 76, P = 0.17) or decay time (Fig. 7E, unexposed: 1.1 ± 0.2 ms, n = 16; exposed: 1.3 ± 0.4 ms, n = 15, Mann-Whitney test: U = 82, P = 0.099). AUT1 did not affect the action potential rise time (Fig. 7B, exposed: 0.4 ± 0.09 ms, n = 15; exposed + AUT1: 0.38 ± 0.06 ms, n = 13, Mann-Whitney test: U = 83, P = 0.99), amplitude (Fig. 7C, exposed: 73.8 ± 11 mV, n = 15; exposed + AUT1: 74.4 ± 8.4 mV, n = 13, Tukey post-hoc test: P = 0.98), half-width (Fig. 7D, exposed: 0.84 ± 0.3 ms, n = 15; exposed + AUT1: 0.73 ± 0.2 ms, n = 13, Mann-Whitney test: U = 75, P = 0.63) or decay time (Fig. 7E, exposed: 1.3 ± 0.4 ms, n = 15; exposed + AUT1: 1.2 ± 0.3 ms, n = 13, Mann-Whitney test: U = 82, P = 0.99).

### 3.7 Inhibitory effects of acoustic over-exposure on the firing frequency

Similarly to TEA, acoustic over-exposure led to a decreased firing frequency in response to high current injections (Fig. 7G–I, at 1.3 nA, unexposed: 91.7 ± 47 Hz, n = 16; exposed: 43.6 ± 45 Hz, n = 16, Mann-Whitney test, U = 55, P = 0.009). AUT1 was ineffective in restoring the firing frequency following acoustic over-exposure (Fig. 7G–I, at 1.3 nA; exposed: 43.6 ± 45 Hz, n = 16; exposed + AUT1: 63.8 ± 48 Hz, n = 14, Mann-Whitney test: U = 84, P = 0.48). Similar firing frequencies were observed in response to low (0.7 nA) current injections after acoustic over-exposure, and in presence of AUT1 following exposure to loud sound (Fig. 7H, unexposed 41.2 ± 26.1: n = 16; exposed: 33.7 ± 31.5 Hz, n = 16, exposed + AUT1: 40.5 ± 29.9 Hz, n = 14, Kruskal-Wallis test P = 0.71), consistent with acoustic over-exposure selectively affecting high voltage activated K^+^ currents in DCN fusiform cells (Pilati et al., 2012).

### 3.8 Effects of acoustic over-exposure on inter-spike interval regularity and spike-time reliability

**Fig. 8:**
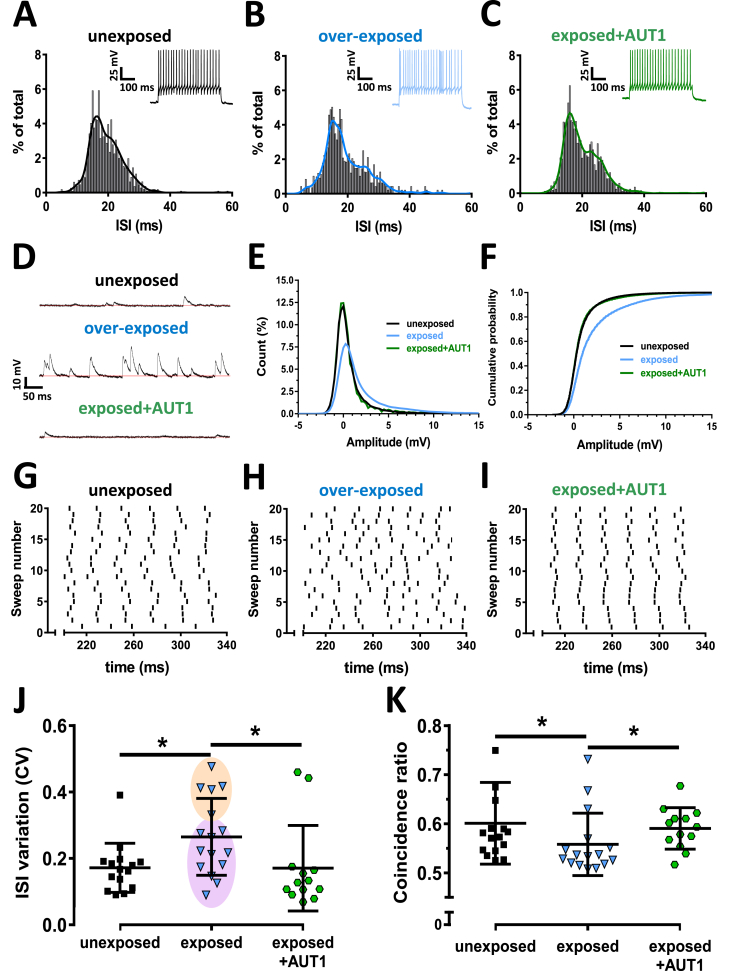
**Effects of positive K**^**+**^**current modulation on inter-spike interval variation and spike coincidence following acoustic over-exposure.** (**A**–**C**) Histograms show the ISI distributions from all cells firing between 25 and 75 Hz for unexposed (**A**, grey, total events = 22235), exposed (**B**, blue, total events = 9520) and exposed + AUT1 (**C**, green, total events = 16610, AUT1) conditions. Insets show example traces. (**D**) Example traces showing membrane voltage in the unexposed (black), acoustic over-exposure (blue) and exposure + AUT1 (green, AUT1) conditions. (**E**) Plot of frequency distribution of amplitudes in the unexposed (n = 16), exposed (n = 16) and exposed + AUT1 (n = 16, AUT1) conditions. Acoustic over-exposure increased the amplitudes of sub-threshold voltage fluctuations, resulting in a shorter, wider distribution in amplitude (**E**, blue). AUT1 prevented the increase in voltage fluctuations, restoring the tall, narrow distribution in amplitude (green), **F:** Cumulative distribution showing a rightwards shift after acoustic over-exposure (Kolmogorov-Smirnov test: k = 0.219, P = <0.001) and a leftwards shift in the presence of AUT1 Kolmogorov-Smirnov test: k = 0.237, P = <0.0001. (Unexposed: black, n = 16; exposed: blue, n = 16, exposed + AUT1: green, n = 14). Data used for the cumulative distributions consisted of 494466 (grey, unexposed), 498784 (light blue, exposed) and 436436 (green, exposed + AUT1) individual baseline points. (**G-I**) Fusiform cells were held at −80 mV and a repeated current stimulus (500 ms) was injected every 6 s. Raster plots show typical examples of spike-timing across 20 trials in single cells recorded in unexposed (**G**), exposed (**H**) and exposed + AUT1 (**I**, AUT1) conditions. (**J**) Plot showing the effects of AUT1 on ISI variation after acoustic over-exposure, as measured using a coefficient of variation. Acoustic over-exposure increased ISI variation (P = 0.027) and AUT1 could prevent this (P = 0.026). Data from the acoustic over-exposure condition were subsequently split into two groups using k-means clustering (shaded areas). The clustering partitioned the data into two clearly distinguishable groups with the higher ISI variation group (orange shaded area) consisting of 31% of the total. (**K**) Plot showing the effects of AUT1 on the coincidence ratio (see methods) after acoustic over-exposure. Acoustic over-exposure caused a decrease in spike-timing coincidence (P = 0.038), an effect prevented by AUT1 (P = 0.04). This can be visualised by the disruption of the raster plot regularity observed in (**H**) and by its return of regularity in exposed + AUT1 shown in **I**. All data are taken from cells firing between 25 and 75 Hz. (**J**–**K**) Error bars = SD. *P < 0.05. (For interpretation of the references to colour in this figure legend, the reader is referred to the Web version of this article.)

**Fig. 9:**
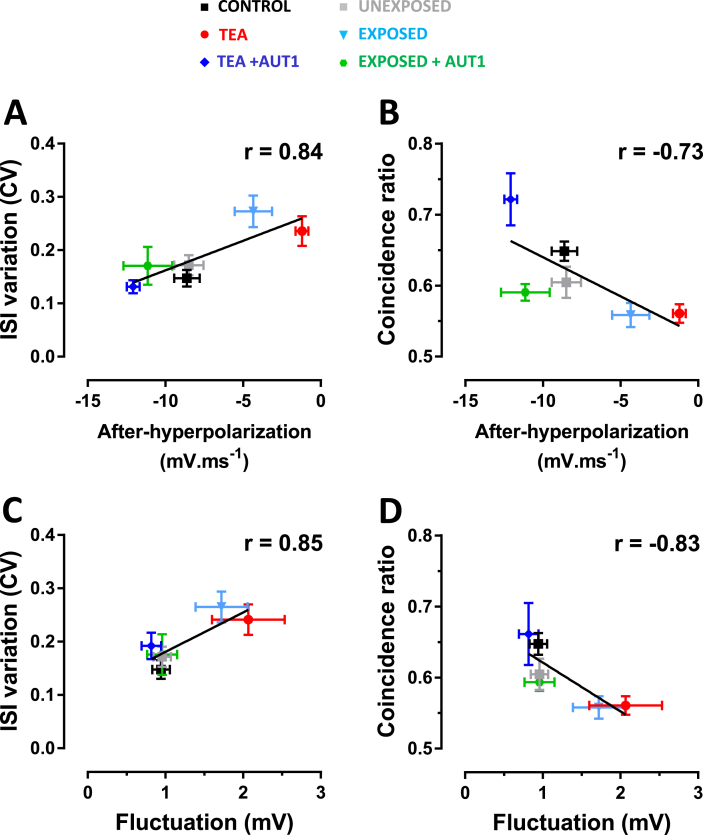
**Correlations between spike-timing and action potential after-hyperpolarization (A,B), and spike-timing and sub-threshold voltage fluctuations (C,D).** Spike-timing was assessed using the inter-spike interval coefficient of variation **(A,C)** and the spike coincidence ratio **(B,D)**. Means ± SEM were obtained from all experimental conditions (control: black; TEA: red; TEA + AUT1: dark blue; unexposed: grey; exposed: light blue; exposed + AUT1: green) and were fitted using a linear regression. Spearman's rho values in the graph are for the mean values (**A**) A significant correlation was observed between ISI variation (CV) and after-hyperpolarization (Spearman's rho = 0.84; Spearman Rho test on individual values r = 0.67, P < 0.0001), coincidence ratio and after-hyperpolarization (**B**, Spearman's rho = −0.73; Spearman Rho test on individual values r = −0.61, P < 0.0001, n = 68), ISI variation (CV) and baseline voltage fluctuation (**C**, Spearman's rho = 0.85; Spearman Rho test on individual values r = 0.52, P < 0.0001, n = 71), and coincidence ratio and baseline voltage fluctuation (**D,** Spearman's rho −0.83; Spearman Rho test on individual values r = −0.53, P < 0.0001, n = 71). (For interpretation of the references to colour in this figure legend, the reader is referred to the Web version of this article.)

Similar to the effects of TEA, acoustic over-exposure disrupted the regular firing pattern (Fig. 8A) of fusiform cells, and resulted in the appearance of burst-like firing (Fig. 8B). This led to a wider distribution of inter-spike intervals (Fig. 8B) and to an increased coefficient of variation of the inter-spike intervals (Fig. 8J, unexposed: 0.17 ± 0.07, n = 15; exposed: 0.26 ± 0.12, n = 16, Mann-Whitney test: U = 58, P = 0.027) compared to unexposed conditions. Previous reports have shown that only a small proportion (∼30–40%) of fusiform cells exhibit a burst firing pattern following acoustic over-exposure (Finlayson and Kaltenbach, 2009, Pilati et al., 2012). We therefore confirmed that the increase in the CV of the inter-spike interval observed following acoustic over-exposure occurred only for a subpopulation of bursting neurons. Inter-spike interval CV data from the acoustic over-exposure condition (Fig. 8J) was partitioned into two groups using k-means clustering. This revealed two clearly distinguishable groups (orange and purple shaded areas in Fig. 8J) and the group with the highest coefficient of variation (orange shaded area in Fig. 8J) consisted of 31% of the total. Exposure to loud sound also increased the amplitudes of membrane potential fluctuations at baseline (Fig. 8D middle, E), resulting in a significant rightwards shift of the cumulative distribution of membrane amplitude counts (Fig. 8F, unexposed: black, n = 16; exposed: blue, n = 16; Kolmogorov-Smirnov test: k = 0.219, P < 0.001). Furthermore, acoustic over-exposure disrupted the ability of fusiform cells to fire reliably across trials (Fig. 8H), resulting in a significant increase in the coincidence ratio (Fig. 8K, unexposed: 0.6 ± 0.08, n = 16; exposed: 0.56 ± 0.06, n = 16, Mann-Whitney test: U = 66, P = 0.038). Similar to its effects in presence of TEA, AUT1 prevented the appearance of irregular, burst-like firing after acoustic over-exposure (Fig. 8C). AUT1 prevented the increase in the coefficient of variation of the inter-spike interval caused by acoustic over-exposure (Fig. 8J, exposed: 0.26 ± 0.12, n = 16; exposed + AUT1: 0.17 ± 0.13, n = 13, Mann-Whitney test: U = 48, P = 0.026). Furthermore AUT1 reduced the baseline membrane potential fluctuations observed after acoustic over-exposure (Fig. 8F exposed: blue, n = 16; exposed + AUT1: green, n = 14; Kolmogorov-Smirnov test: k = 0.237, P = <0.0001), and restored reliable spike-timing (Fig. 8I), as measured using the coincidence ratio (Fig. 8K exposed: 0.56 ± 0.06, n = 16; exposed + AUT1: 0.59 ± 0.04, n = 13, Mann-Whitney test: U = 51, P = 0.0392). As both TEA and acoustic over-exposure affected firing reliability concomitantly with changes in the action potential after-hyperpolarization and in the level of membrane potential fluctuations, we finally tested whether spike-timing was correlated with those two factors. A strong correlation was observed between the action potential after-hyperpolarization and the inter-spike interval coefficient of variation (r = 0.84, Fig. 9A), and between the action potential after-hyperpolarization and reliable spike timing assessed using the coincidence ratio (r = −0.73, Fig. 9B). A strong correlation was also observed between the amplitudes of membrane potential fluctuations and the inter-spike interval coefficient of variation (r = 0.85, Fig. 9C), and between membrane potential fluctuation amplitudes and the coincidence ratio (r = −0.83, Fig. 9D).

## 4 Discussion

Pilati et al. (2012) reported that a down-regulation of K^+^ currents with similar biophysical properties to those mediated by Kv3 K^+^ channels was associated with a disruption of spike-timing of DCN fusiform cells following acoustic over-exposure. The present study investigated the effects of TEA and acoustic over-exposure on fusiform cell action potential characteristics and firing properties. We used AUT1, a novel modulator of human and rodent Kv3.1 and Kv3.2 K^+^ channels (Alvaro et al., 2011, Brown et al., 2016, Rosato-Siri et al., 2015, Taskin et al., 2015) to evaluate further the contribution of Kv3 K^+^ channels to fusiform cell firing properties following pharmacological reduction of K^+^ currents by TEA or following acoustic over-exposure. In particular, we explored the effects on the firing frequency and on the spike-timing (by measuring the regularity of the inter-spike interval and the ability to fire consistently across repetitive applications of a stimulus (Joris et al., 2006, Street and Manis, 2007). We also tested whether the modulation of the spike-timing was dependent on an effect on the action potential after-hyperpolarization (Erisir et al., 1999, Wang et al., 1998) and on spontaneous membrane voltage fluctuations (Dorval and White, 2005, Jaeger and Bower, 1999).

Our study shows that TEA (Fig. 4) and acoustic over-exposure (Fig. 7) reduce the maximum firing frequency in DCN fusiform cells (Pilati et al., 2012), and that AUT1 failed to prevent this effect (Fig. 4, Fig. 7). This is in contrast with the ability for the drug to prevent the effects of TEA on maximal firing frequencies (>100 Hz) in parvalbumin-positive cortical interneurons (Rosato-Siri et al., 2015). The question arises whether the firing frequency of DCN fusiform cells is controlled by other types of K^+^ currents blocked by TEA. Indeed TEA blocks Kv3 K^+^ currents (Hernandez-Pineda et al., 1999, Wang et al., 1998), but also Kv1.1 and Kv7.2 K^+^ currents (Al-Sabi et al., 2013, Johnston et al., 2010, Li et al., 2013, Scott et al., 2003) and BK Ca^2+^ dependent K^+^ currents (Krause et al., 1996). In the present study, TEA only decreased the firing frequency in response to high current injections (Fig. 4B–D), suggesting that its effect is unlikely to be due to the inhibition of low threshold, rapidly inactivating Kv1.1 K^+^ currents (Brew and Forsythe, 1995, Pilati et al., 2012). Kv7.2/3 K^+^ are also implicated in tinnitus and noise exposure has been shown to decrease Kv7.2/3 K^+^ currents (Li et al., 2013, Li et al., 2015). However blocking these channels has been shown to increase rather than decrease the firing frequency (Battefeld et al., 2014, Guan et al., 2011). Alternatively, inhibition of BK Ca^2+^ dependent K^+^ channels has been reported to increase (Nelson et al., 2003), decrease (Gu et al., 2007, Lin et al., 2014) or have no effect (Kimm et al., 2015) on the firing frequency. It is therefore possible that the decrease of firing frequency observed following the addition of TEA or acoustic over-exposure is due an action on BK K^+^ channels, although the absence of effect of AUT1 on the firing frequency suggests that Kv3 K^+^ channels are not involved in this process.

Our study confirms that spike-timing is an important aspect of information coding in the DCN, as previously demonstrated (Street and Manis, 2007), and that TEA (Fig. 5, Fig. 6) or acoustic over-exposure (Fig. 8) lead to spike-timing disruption of DCN fusiform cells (Finlayson and Kaltenbach, 2009, Pilati et al., 2012). We further show that AUT1 applied in the presence of TEA, or after acoustic over-exposure, counteracted the disruptive effects on the inter-spike regularity (Fig. 5, Fig. 8 C,J) and the spike-timing reliability (Fig. 6, Fig. 8K). It is noteworthy that a previous study identified a bursting pattern in about 30% of rat DCN fusiform cells (Pilati et al., 2012). We confirmed that about 30% of the fusiform cells displayed a high coefficient of variation (Fig. 8J, orange shaded area), and therefore can be assimilated to bursting neurones.

Previous studies have shown that TEA (Erisir et al., 1999, Wang et al., 1998) and acoustic over-exposure (Pilati et al., 2012) reduced the amplitude of DCN fusiform cell action potential after-hyperpolarization. Our study confirms these effects (Fig. 3, Fig. 7F) and shows that the disruptive effect on spike-timing, in the presence of TEA or after acoustic over-exposure is related to the reduced action potential after-hyperpolarization (Fig. 9A, B). We further showed that AUT1 applied in presence of TEA, or after acoustic over-exposure enhanced the action potential after-hyperpolarization amplitude (Fig. 3, Fig. 7F). Under normal conditions, Kv3 K^+^ channels typically activate at voltages more depolarized than −20 mV (Johnston et al., 2010, Rudy and McBain, 2001). AUT1 shifts the open probability of Kv3 channels towards action potential threshold values (Brown et al., 2016, Rosato-Siri et al., 2015, Taskin et al., 2015), leading to KV3 K^+^ channels activating earlier during the rising phase of an action potential, and deactivating later during the action potential repolarization. This is consistent with AUT1 increasing the action potential after-hyperpolarization amplitude and provides an explanation for the increased after-hyperpolarization amplitude when comparing AUT1 (in presence of TEA or after acoustic over exposure) with control or unexposed conditions. Cross-screening experiments showed that AUT1 is specific for Kv3.1 and Kv3.2 channels over a wide range of ion channels, receptors, and transporters (Table 1, Rosato-Siri et al., 2015). We propose that AUT1 enhances the after hyperpolarization by increasing Kv3 mediated K^+^ currents. However, it is possible that other, as yet unknown pharmacological interactions of AUT1 could contribute to the observed effects in this study.

We tested whether baseline membrane voltage fluctuations arising of synaptic origin were a limiting factor in a fusiform cell's ability to fire with precision (Jaeger and Bower, 1999, Street and Manis, 2007). TEA and acoustic over-exposure increased the amplitude of baseline membrane voltage fluctuations (Fig. 6, Fig. 8F), decreased spike-timing precision (Fig. 6, Fig. 8) and spike-timing reliability (Fig. 6, Fig. 8K). These results are in accordance with an increase in spontaneous activity within the DCN (Brozoski et al., 2002, Kaltenbach et al., 1998) and an increased release probability at glutamatergic multisensory synapses (Tagoe et al., 2017) following acoustic over-exposure. AUT1 prevented the up-regulation of baseline membrane voltage fluctuations when added in the presence of TEA (Fig. 6A–C) or after acoustic over-exposure (Fig. 8D–F). AUT1 also restored spike-timing precision (Fig. 6, Fig. 8) and spike-timing reliability (Fig. 6, Fig. 8K). This could be due to the modulation of Kv3 K^+^ channels expressed on presynaptic neurons (most likely at the pre-synaptic bouton, where the channels regulate calcium entry and transmitter release). For example DCN granule cells (Perney and Kaczmarek, 1997) cartwheel cells (Roberts and Trussell, 2010), or neurons in neighbouring brainstem regions such as the ventral cochlear nucleus (Doucet and Ryugo, 1997, Rusznak et al., 2008) all send synaptic projections onto DCN fusiform cells. Our study shows that the spike-timing is correlated to the amplitude of the membrane potential fluctuations of synaptic origin (Fig. 9C, D). Bursts of action potential can still be observed after blocking synaptic transmission in the presence of TEA (Fig. 6C), showing that membrane potential fluctuations are not the sole factor involved in modulating spike-timing. In this instance (Fig. 6C) bursts could be due to the reduced after-hyperpolarization lowering threshold potentials and shortening refractory periods (Chen et al., 2006).

The present results are consistent with a reduced Kv3 K^+^ current in fusiform cells following acoustic over-exposure or TEA (Pilati et al., 2012), and the modulation of spike-timing in DCN fusiform cells due to an action on the action potential hyperpolarization and on membrane voltage fluctuations of synaptic origin. Although fusiform cells display almost no phase locking for frequencies exceeding 1 kHz (Rhodes and Smith, 1986), studies have demonstrated the importance of temporal precision in spike-timing dependent plasticity evoked at parallel fibre synapses onto fusiform cells (Tzounopoulos et al., 2004). As DCN fusiform cells integrate acoustic with somatosensory information (Wu et al., 2016), it is possible that modulation of spike-timing could modify the response to spectral cues and/or modifies the perception profiles of sensory stimuli or the encoding of speech components (Presacco et al., 2015).
